# *In situ* SERS reveals nickel hydroxide formation in PtRuNi catalysts enhances hydrogen oxidation

**DOI:** 10.1039/d6na00164e

**Published:** 2026-05-15

**Authors:** Kai Tao, Mingxiao Han, Li Jiang, Shangzhong Jin, Tommaso Giovannini, Denis Garoli, Zhefei Zhao, Qiang Lin, Huaizhou Jin

**Affiliations:** a Key Laboratory of Quantum Precision Measurement, School of Physics and Optical Engineering, Zhejiang University of Technology Hangzhou 310014 China jinhuaizhou@zjut.edu.cn; b College of Optical and Electronic Technology, China Jiliang University Hangzhou 310018 China; c Department of Physics, University of Rome Tor Vergata, INFN Via della Ricerca Scientifica 1 I-00133 Rome Italy; d Dipartimento di Scienze e Metodi dell’Ingegneria, Università degli Studi di Modena e Reggio Emilia Viale Amendola 2 Reggio Emilia Italy denis.garoli@unimore.it; e Department of Applied Chemistry, Petroleum and Chemical Industry Key Laboratory of Organic Electrochemical Synthesis, State Key Laboratory of Green Chemical Synthesis and Conversion, Zhejiang University of Technology Hangzhou 310014 China; f State Key Laboratory of Ocean Sensing, Institute of Quantum Sensing, School of Physics, Zhejiang University Hangzhou 310058 China

## Abstract

Understanding the mechanisms for improving hydrogen oxidation reaction (HOR) activity on Pt-based catalysts in alkaline environments is challenging due to the lack of directly observed intermediates. Understanding the role of oxophilic metal additives is also crucial for designing advanced catalysts. In this study, we use *in situ* surface-enhanced Raman spectroscopy (SERS) to investigate the HOR on Au@PtRuNi nanoparticles. Electrochemical measurements show improved HOR activity with little change in hydrogen binding energy (HBE), whereas Raman results show the 740 cm^−1^ peak for hydroxyl adsorption on Ru, as well as a new peak at 565 cm^−1^, which can likely be attributed to the formation of nickel hydroxide (Ni(OH)_2_) during the catalytic process, starting from 0.3 to 0.5 V *vs.* RHE. This suggests that nickel hydroxide and the hydroxyl groups on ruthenium likely collaborate to facilitate hydrogen oxidation.

## Abstract 2

Pt-based catalysts in alkaline media show enhanced HOR activity. SERS reveals that Ru–OH and Ni(OH)_2_ species synergistically promote hydrogen oxidation.

## Graphical abstract

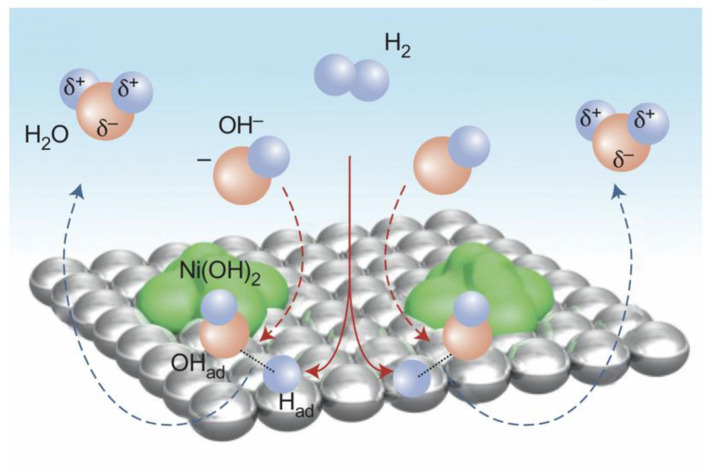

## 1. Introduction

Hydrogen fuel cells are a promising technology for green energy generation; they are highly efficient and produce only water as a byproduct, making them environmentally friendly.^1–4^ One critical half-reaction in hydrogen fuel cells is the hydrogen oxidation reaction (HOR). In fact, the efficiency of this reaction is significantly influenced by the electrocatalyst.^5–8^ Platinum (Pt) is a well-known electrocatalyst in the HOR; however, because of its scarcity and high cost, researchers are actively exploring other metals and alloys to enhance catalytic activity and reduce costs.^9–11^ In recent years, alkaline anion exchange membrane fuel cells (AEMFCs) have gained significant attention due to their ability to work better with non-platinum group metal catalysts.^12–15^ However, the HOR is significantly slower in alkaline media than in acid. Therefore, researchers are trying to develop new catalysts that can operate efficiently in alkaline environments.^16^ Among them, ruthenium (Ru) and nickel (Ni) are two particularly promising candidates for enhancing Pt-based catalyst performance.^17–19^ Studies have also shown that multi-metal catalysts, such as those formed by implanting oxophilic metals into PtRu structures, can enhance HOR activity; however, their mechanisms have not yet been fully elucidated.^20^

To understand the HOR in alkaline media, we first start with its reaction equation. The HOR in alkaline media consists of a few basic steps: Tafel, Heyrovsky, and Volmer steps, with the first step being the Tafel or Heyrovsky process, and the second being the Volmer process.^17,21^ The reaction equations of Tafel, Heyrovsky and Volmer steps are shown in (1)–(3), with the total reaction equation shown in eqn (4):

1 Tafel step: * + H_2_ → 2H_ad_

2 Heyrovsky step: * + H_2_ + OH^−^ → H_ad_ + H_2_O + e^−^

3 Volmer step: H_ad_ + OH^−^ → * + H_2_O + e^−^

4 Total equation: 2* + H_2_ + 2OH^−^ → 2* + 2H_2_O + 2e^−^

In these equations, * represents hydrogen adsorption sites. Heyrovsky and Volmer steps involve the participation of hydroxyl (OH) species. The source of OH^−^ species and its precise role remain areas of active investigation and debate.^22,23^ The two dominant mechanistic proposals are the hydrogen binding energy (HBE) theory and the bifunctional mechanism.^24^ Supporters of HBE theory emphasize that the hydrogen binding energy is the primary factor that affects HOR kinetics.^25^ On the other hand, supporters of the bifunctional mechanism postulate that HOR kinetics in alkaline media is limited by the Volmer step, and that adsorbed OH species on oxophilic metal or compound sites on the electrocatalysts would enhance HOR activity by facilitating the Volmer step.^26^ One of the earliest supporters of the bifunctional mechanism, Nenad M. Marković's group, successfully modified Pt(111) with Ni(OH)_2_-islands, and suggested that adsorbed OH species are a key reactant in the HOR in alkaline solutions.^27^ One critical reason for this debate stems from the technical challenges in directly observing the short-lived hydroxyl intermediates during the catalysis.^28–30^ Surface-enhanced Raman spectroscopy (SERS) is a powerful tool for identifying and characterizing these transient species at the electrode–electrolyte interface (*i.e. in situ*).^31^ This technique can be employed to identify adsorbed hydroxyl species and other intermediates, providing direct spectroscopic evidence to elucidate their role in the reactions.^5^ In several previous studies, Ru has been identified as a key oxophilic metal for OH adsorption, with the metal's ability to adsorb OH species at very low potentials.^32,33^ Specifically, SERS results indicated that the formed ruthenium oxides served as robust OH adsorption sites, thereby facilitating the HOR process.^33^ Ni can also provide OH adsorption sites, but the adsorption may happen at a higher potential.^34^

**Fig. 1:**
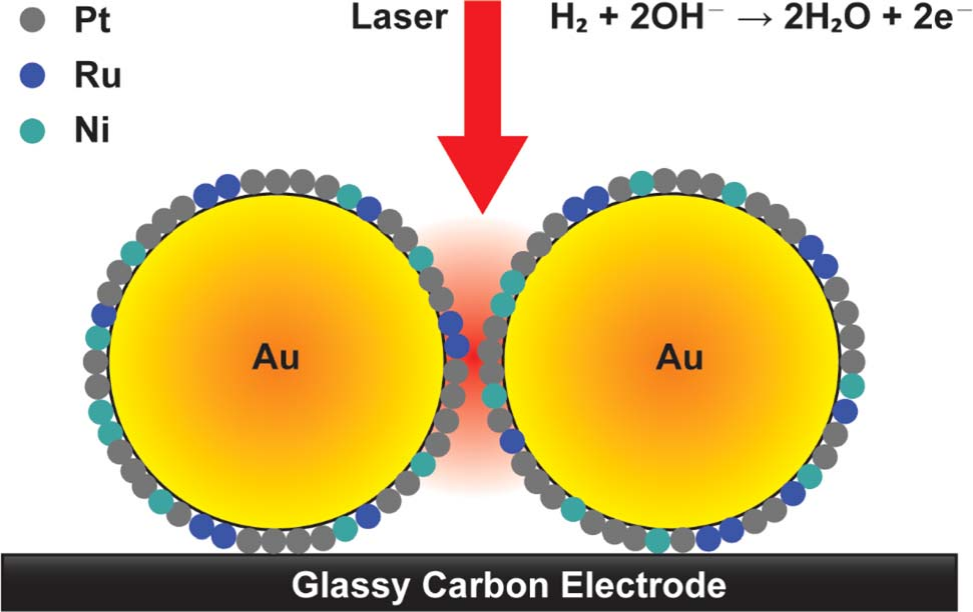
Illustrative schematic of the HOR process on the Au@PtRuNi surface in alkaline media. (The choice for the size of the Au core and Pt, Ru and Ni composition is related to the composition measured with EDS).

In this study, we aim to utilize SERS to investigate the behavior of adsorbed hydroxyl intermediates on Pt and PtRu based catalysts by using Au@Pt, Au@PtRu and Au@PtRuNi core–shell structures.^35^ The Au@PtRuNi structure is used to investigate the role of Ni in the HOR process, and that whether Ni can act in synergy with Ru to improve HOR activity. The Au@PtRuNi core–shell structure is shown in Fig. 1. This comparative analysis with multiple catalysts using both electrocatalytic measurement and SERS allows for a nuanced understanding of how different oxophilic elements influence the interfacial interactions in the HOR, and allows us to discover possible nickel hydroxide formation which facilitates the HOR process.

## 2. Materials and methods

### 2.1 Materials and instrumentation

Gold chloride trihydrate (HAuCl_4_·3H_2_O, 99.9%) is the precursor used to synthesize gold nanoparticles (AuNPs). Metal precursors used for catalysis synthesis include chloroplatinic acid hexahydrate (H_2_PtCl_6_·6H_2_O, 99.9%), ruthenium(iii) chloride hydrate (RuCl_3_·*x*H_2_O), and nickel chloride hexahydrate (NiCl_2_·6H_2_O).

Reductive agents including sodium borohydride (NaBH_4_), ascorbic acid and sodium citrate were employed for the synthesis; ethanol, potassium hydroxide, and ultrapure water (18.2 MΩ cm) were used for solution preparation and cleaning procedures.

All reagents above were purchased from various domestic providers, such as Adamas-beta, Macklin and Aladdin, all from Hangzhou and Shanghai, China. They were all of analytical grade and were used without further purification. Vulcan carbon XC-72 was utilized as the catalyst support material, obtained from Cabot Corporation, Boston, United States.

Electrochemical measurements were performed using a DHElecchem electrochemical workstation with a standard three-electrode setup, using the built-in DHElecchem software to record electrochemical data. A Gauss Union C031-4 *in situ* electrochemical flow cell was used for all electrochemical and *in situ* Raman measurements. The flow cell was equipped with a standard tri-electrode setup, including a platinum wire counter electrode, a mercury/mercurous oxide (Hg/HgO) reference electrode, and a glassy carbon working electrode, with all potentials referenced against the reversible hydrogen electrode (RHE) in 0.1 M KOH solution.

For Raman measurements, we used a Horiba XploRA Raman Spectrometer (Horiba Jobin Yvon, Paris, France). A 638 nm laser was used as the excitation laser. Strong fluorescent interference was present for SERS measurements when a 532 nm laser was used; on the other hand, the signals we obtained when using a 785 nm laser were too weak.

### 2.2 Methods

#### 2.2.1 Synthesis of 55 nm AuNPs

55 nm AuNPs were synthesized according to Frens' method.^36,37^ Specifically, 2.424 mL of 0.825% HAuCl_4_ aqueous solution was combined with 196 mL of water in a three-necked round-bottom flask. This mixture was subsequently brought to a boil, followed by the rapid addition of 1.5 mL of 1% trisodium citrate solution under continuous stirring while boiling for 20 minutes. The resulting colloidal solution was then cooled to room temperature and stored for further use.

#### 2.2.2 Synthesis of Au@Pt nanoparticles

Core–shell Au@Pt nanoparticles were synthesized following similar steps as reported in previous chemical reduction methods.^33,34^ Specifically, Au@Pt nanoparticles were synthesized by first combining 30 mL of gold colloidal solution with 0.72 mL of 1 mM aqueous H_2_PtCl_6_; this mixture was then stirred at 80 °C, followed by the dropwise addition of 0.36 mL of 10 mM aqueous ascorbic acid using a step motor-controlled syringe. Stirring was maintained for an additional 20 minutes after the complete addition.

#### 2.2.3 Synthesis of Au@PtRu nanoparticles

After the synthesis of Au@Pt nanoparticles, Ru was reduced on the surface by adding 120 µL of 1 mM RuCl_3_ and 60 µL of 1% trisodium citrate quickly while the solution was boiling. The solution was then stirred while boiling for another 20 minutes to complete the reduction process, forming Au@PtRu.

#### 2.2.4 Synthesis of Au@PtRuNi nanoparticles

For Au@PtRuNi nanoparticles, Pt and Ni were reduced on the surface of AuNPs simultaneously by combining 30 mL of gold colloidal solution with 0.72 mL of 1 mM aqueous H_2_PtCl_6_ and 0.72 mL of 1 mM aqueous NiCl_2_ solution. This mixture was then stirred at 80 °C, followed by the dropwise addition of 0.72 mL of 10 mM aqueous ascorbic acid using a step motor-controlled syringe, and stirring was maintained for an additional 20 minutes after the complete addition. Then, Ru was reduced on the surface using exactly the same method in the synthesis of Au@PtRu nanoparticles.

After the synthesis of these Au@catalyst core–shell nanoparticles, transmission electron microscopy (TEM) was used to characterize their morphology and size distribution, with elemental composition and mapping characterized by energy-dispersive X-ray spectroscopy (EDS). Additionally, electrochemical techniques such as cyclic voltammetry (CV) and linear sweep voltammetry were employed to evaluate the catalytic activity with the aforementioned DHElecchem electrochemical workstation. *In situ* Raman spectroscopy measurements were conducted under constant potential conditions (such as −0.015 V, 0.035 V, 0.085 V, 0.135 V, *etc.*) to investigate intermediates during the electrochemical processes.

## 3. Results

### 3.1 Characterization of nanoparticles

**Fig. 2:**
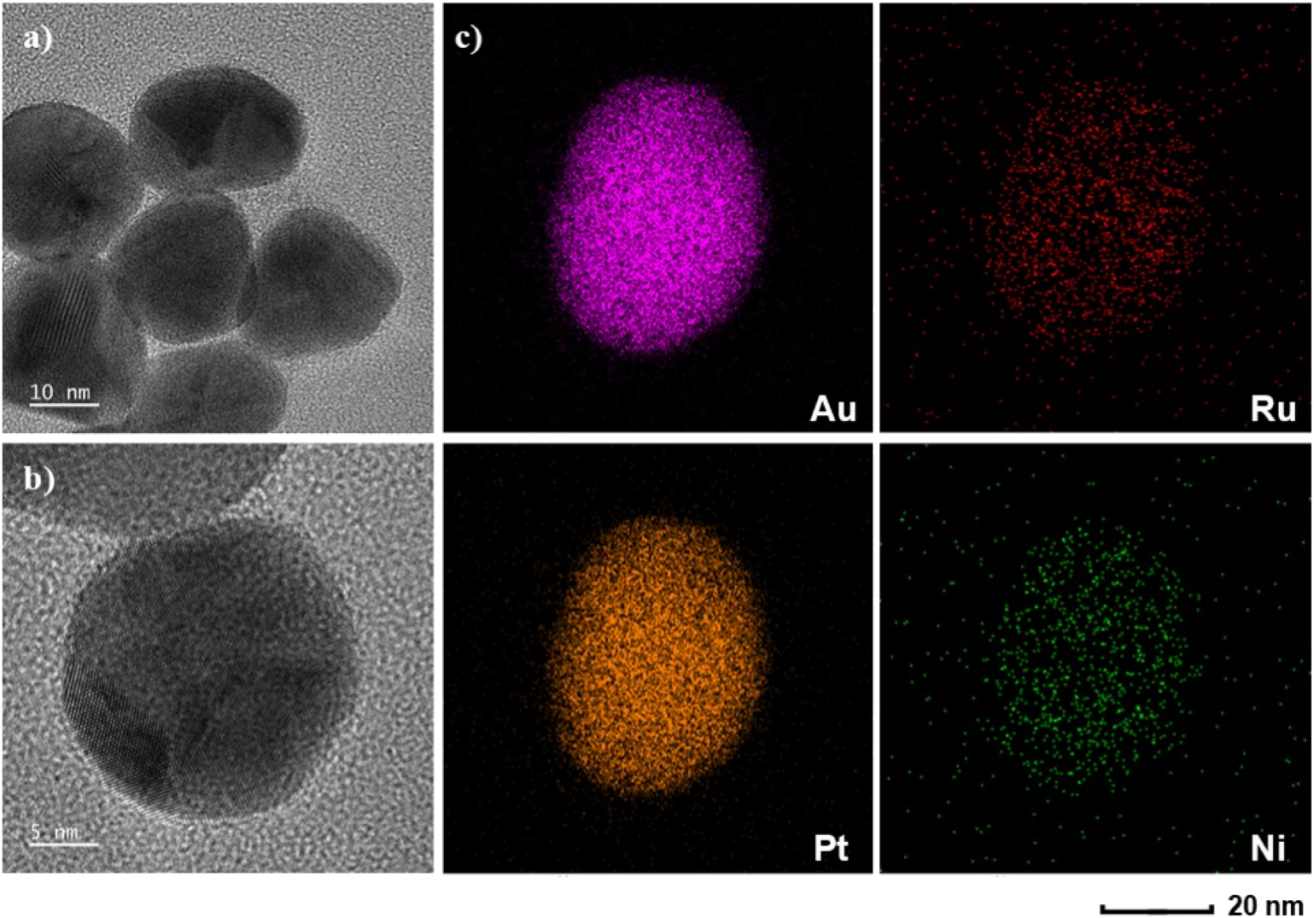
Characterization of Au@PtRuNi nanoparticles. (a) The TEM results of nanoparticles; scale bar: 10 nm; (b) the TEM results of one nanoparticle; scale bar: 5 nm; (c) elemental mapping from one single nanoparticle for Au, Pt, Ru and Ni, respectively; scale bar: 20 nm.

Fig. 2 shows the characterization results of Au@PtRuNi nanoparticles. Fig. 2(a) and (b) show the TEM result of the nanoparticles. The TEM images suggest that the chemical reduction method produced uniform nanoparticles with an average diameter of 30 nm and well-defined morphology.

**Fig. 3:**
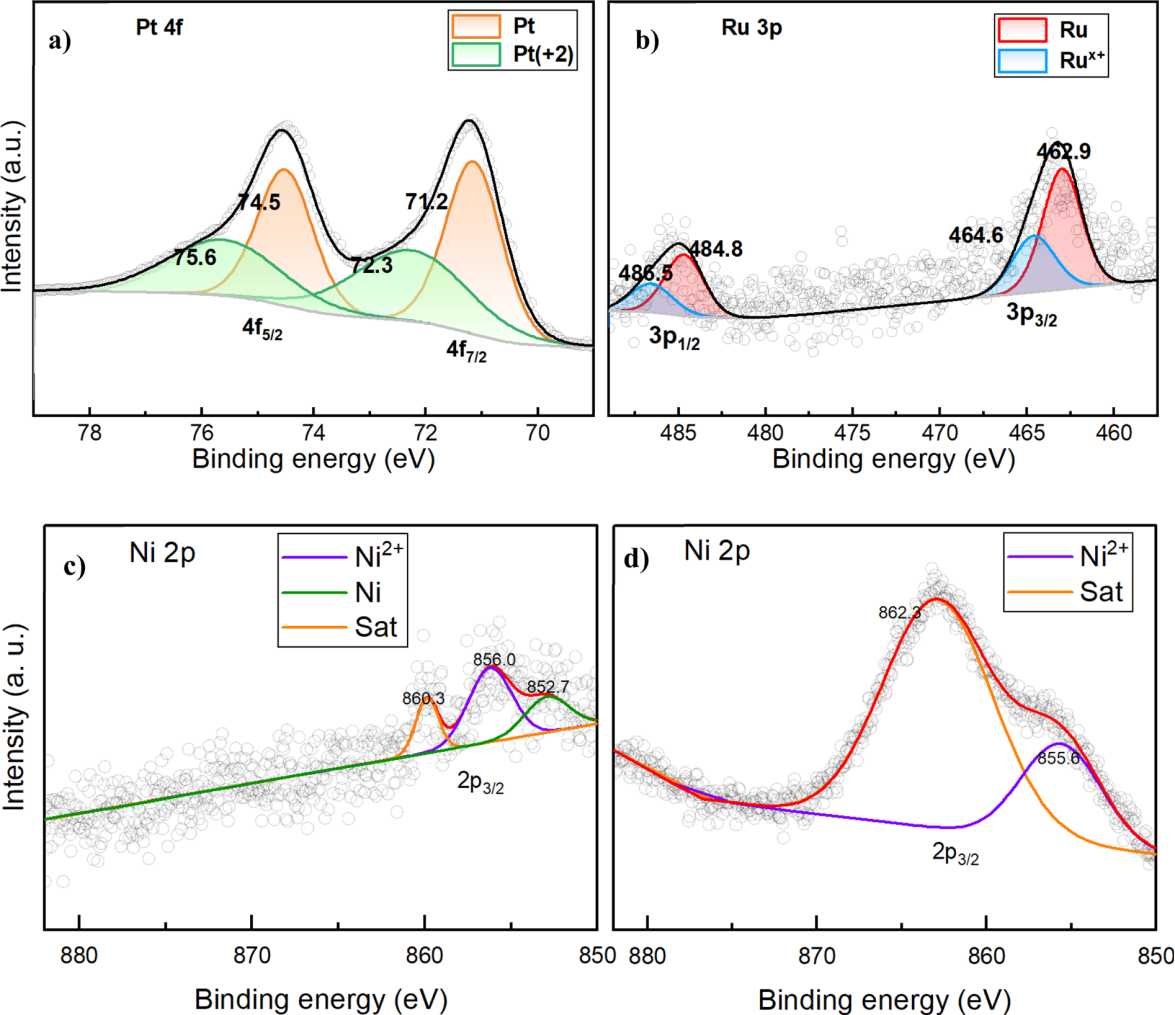
(a and b) XPS results of Pt 4f and Ru 3p of Au@PtRuNi nanoparticles, respectively; (c) XPS result of Ni 2p of Au@PtRuNi nanoparticles, before the HOR; (d) XPS result of Ni 2p of Au@PtRuNi nanoparticles, after the HOR.

EDS elemental mapping reveals the spatial distribution of Au, Pt, Ru, and Ni throughout the nanoparticles. Fig. 2(c) shows the distribution of the four individual metals. The Au and Pt signals are concentrated in the center of the figure, this suggests more continuous distribution of Au and Pt, whereas the Ru and Ni signals are spread more sparsely over the nanoparticle. Quantitative EDS analysis of Au, Pt, and Ru shows atomic percentages of 77.80%, 21.01%, and 1.18%, respectively (normalized for Au, Pt and Ru only). Nickel content could not be accurately quantified by EDS; however, the presence of nickel content is confirmed in XPS results in Fig. 3(c) and (d). In fact, X-ray photoelectron spectroscopy (XPS) was used to characterize the surface chemical states of the Au@PtRuNi nanoparticles, as shown in Fig. 3. The Pt 4f spectrum displays two prominent peaks at binding energies of 71.2 eV (Pt 4f_7/2_) and 74.5 eV (Pt 4f_5/2_), which correspond to metallic Pt, with a minor shoulder at ∼72.3 eV suggesting the presence of partially oxidized Pt species Pt(ii). The Ru 3p_3/2_ peak is observed at 462.9 eV, consistent with metallic Ru, while the broadening toward higher binding energy indicates a small fraction of RuO_*x*_, which is in agreement with the role of Ru as an oxophilic site for OH adsorption. XPS spectra of Ni 2p are taken both before and after the HOR. In Fig. 3(c), two main Ni 2p_3/2_ peaks appear at 856.0 eV and 852.7 eV corresponding to Ni(ii) and metallic Ni, respectively. XPS results further show that the deposition of Pt, Ru and Ni on the Au core is successful. In addition, as shown in Fig. 3(d), the XPS curve changes after HOR electrocatalysis. The 852.7 eV peak corresponding to metallic Ni disappears while the 855.6 eV peak, which corresponds to Ni(ii), becomes the main nickel peak in the spectrum. The shift of the satellite peak from 860.3 to 862.3 eV also suggests that nickel species are oxidized during the HOR process.

### 3.2 Electrocatalytic performances

**Fig. 4:**
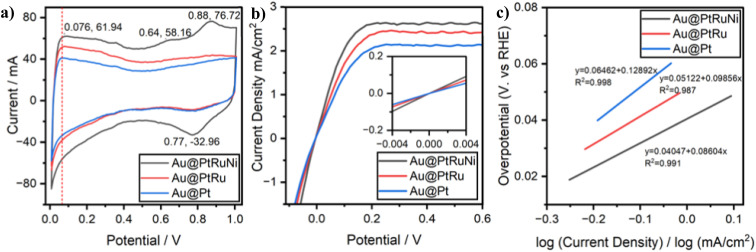
(a) Cyclic voltammetry curves of Au@Pt, Au@PtRu and Au@PtRuNi, measured in a potential range from 0.05 to 1.0 V *vs.* RHE at a scan rate of 10 mV s^−1^ in 0.1 M KOH solution saturated with H_2_; (b) polarization curve obtained from linear sweeping voltammetry from −0.1 to 0.6 V *vs.* RHE at a scan rate of 10 mV s^−1^ and 1600 rpm rotation speed, inset is the micro-polarization region from −4 to 4 mV; (c) Tafel plots derived from HOR polarization curves using normalized kinetic current densities.

Fig. 4(a) shows the HOR cyclic voltammetry (CV) curves of the HOR on Au@Pt, Au@PtRu and Au@PtRuNi nanoparticles. CV was performed in the range from 0.0 to 1.0 V *vs.* RHE at a scan rate of 10 mV s^−1^ in 0.1 M KOH solution saturated with H_2_. On all CV curves, we can see hydrogen underpotential deposition (H_upd_) peaks around 76 mV. This means that the addition of oxophilic metals on the Pt layer did not alter the hydrogen binding energy (HBE) significantly. On the CV curve of Au@PtRuNi, there are exclusive metal redox peaks at 0.77 and 0.88 V. According to Hall *et al.* and Oshchepkov *et al.*, redox peaks in this region can be assigned to the transition between α-Ni(OH)_2_ and β-Ni(OH)_2_.^38–40^

The CV curves in Fig. 4(a) also show that Au@PtRuNi exhibits a higher double-layer capacitance (*C*_dl_) compared to Au@PtRu and Au@Pt; the increased *C*_dl_ may arise from enhanced surface roughness and/or hydrophilicity from Ni(OH)_2_ formation.

In order to assess the catalytic performance of the three catalysts, we also performed polarization experiments. Fig. 4(b) shows the polarization curves of Au@Pt, Au@PtRu and Au@PtRuNi catalysts obtained from linear sweeping voltammetry in 0.1 M KOH with saturated H_2_ at a sweeping rate of 10 mV s^−1^ and rotating speed of 1600 rpm, with the inset of Fig. 4(b) showing the micro-polarization regions of −4 to 4 mV. Fig. 4(c) shows the Tafel plot obtained from the LSV data from Fig. 4(b). Current densities increase faster for Au@PtRuNi than for Au@PtRu and Au@Pt, with Au@Pt having the slowest increasing of current density. At a fixed current density of 1 mA cm^−2^, Au@PtRuNi requires an overpotential of 38.9 mV, compared to 52.5 mV for Au@PtRu and 65.5 mV for Au@Pt. This 13.6 mV and 26.6 mV improvement over Au@PtRu and Au@Pt, respectively, demonstrates that the catalytic enhancement arises from electronic and chemical effects of Ni(OH)_2_, rather than the increase of surface area and hydrophilicity alone.

These results demonstrate that the inclusion of Ni in the catalyst provides genuine catalytic enhancement. The mechanisms of this enhancement are revealed by *in situ* SERS analysis in the next section.

### 3.3 SERS measurement results

**Fig. 5:**
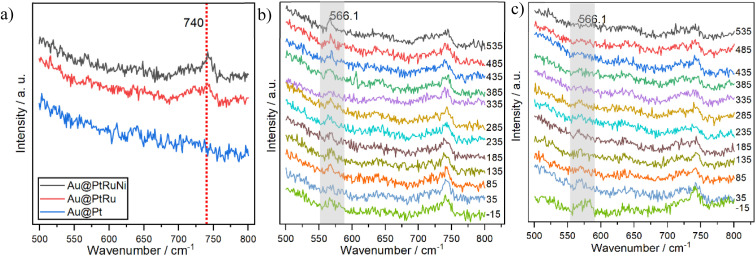
(a) Comparison between SERS spectra of the HOR in alkaline on different catalysts at 185 mV *vs.* RHE potential; (b and c) the trend of SERS spectra of the HOR in alkaline media on Au@PtRuNi and Au@PtRu catalysts, respectively, at different potentials from −15 to 535 mV *vs.* RHE. The potentials (mV) at which the Raman spectra are taken are labeled to the right of each spectra.

We further examine the catalytic mechanisms using *in situ* electrochemical SERS. Fig. 5(a) shows a comparison between the SERS spectra obtained from Au@Pt, Au@PtRu, and Au@PtRuNi alloy nanoparticles at a potential of 0.155 V. Compared to Au@Pt NPs, Au@PtRu and Au@PtRuNi nanoparticles have a clear additional peak at 740 cm^−1^. This peak was reported in the literature and was attributed to OH_ad_ species adsorbed on Ru or RuO_*x*_.^33^ Au@PtRu and Au@PtRuNi have the same 740 cm^−1^ peak, which indicates that Ru or RuO_*x*_ is a primary OH adsorption site.

The changes to the spectra of the HOR on Au@PtRuNi nanoparticles from −0.015 V to 0.535 V (−15 to 535 mV) *vs.* RHE are shown in Fig. 5(b). In these experiments, we also found another peak at 566 cm^−1^ that becomes stronger as potential increases. In the Au@PtRu results shown in Fig. 5(c), a peak is also observed near 566 cm^−1^. However, the 566 cm^−1^ peak obtained from the Au@PtRu catalyst becomes weaker as the potential increases, and disappears before 0.2 V, which is attributed to the oxidation of RuO_*x*_ and is consistent with previous results.^33^ On the other hand, for experiments on Au@PtRuNi nanoparticles, the intensity of 566 cm^−1^ increases as the potential increases, especially at and above 0.385 V *vs.* RHE. Hence, the increasing 566 cm^−1^ peak on Au@PtRuNi nanoparticles is associated primarily with Ni compounds. Nickel has two primary (oxy)hydroxide species in electrochemistry: nickel hydroxide, Ni(OH)_2_, where Ni is predominantly in the +2 oxidation state, or Ni(ii), and nickel oxyhydroxide, NiOOH, which contains nickel in the +3 oxidation state, or Ni(iii). Our post-reaction XPS result in Fig. 3(d) shows a prominent Ni(ii) peak at 855.6 eV; the metallic Ni peak, which is present in the pre-reaction XPS curve in Fig. 3(c) and disappears in Fig. 3(d), suggesting that most Ni species are oxidized. We performed peak fitting on the post-reaction XPS result, and the fit could not converge when a Ni(iii) peak was added. This suggests that the contribution of Ni(iii) is non-existent or extremely minimal, and that Ni(ii) is the dominant nickel species in the HOR process. In the literature, the formation of NiOOH or Ni(iii) typically requires higher potentials; 1.2 V to 1.4 V *vs.* RHE are the minimum potentials required for NiOOH formation, whereas Ni(OH)_2_ can form at lower potentials, such as at 0.5 V *vs.* RHE.^41,42^ According to recent studies, the transition of α-Ni(OH)_2_ to β-Ni(OH)_2_ can take place at low potentials, which is a phenomenon consistent with the two distinct oxidation peaks observed in the cyclic voltammetry curve of Au@PtRuNi; moreover, Hall *et al.* assigned the peak around 566 cm^−1^ to β-Ni(OH)_2_, and α-Ni(OH)_2_ exhibits no such peaks near that region.^38,43^ Xue *et al.* also showed that in a RuNi/C catalyst, Ni species are present as oxidized Ni(ii) under operating conditions.^44^ Given these pieces of evidence, we attribute the 566 cm^−1^ peak on Au@PtRuNi to Ni(OH)_2_. The distinct behavior of the 566 cm^−1^ peak in the Au@PtRuNi system, intensifying with increasing potential, suggests the potential involvement of nickel hydroxide species that may enhance the HOR catalytic activity.

We also performed peak-fitting for the 740 cm^−1^ peak on Au@PtRuNi and Au@PtRu catalysts; the results (SI Fig. S2) show that the area of the 740 cm^−1^ peak on Au@PtRuNi is larger at lower potentials and gradually decreases as the potential increases, whereas the 740 cm^−1^ band on Au@PtRu remains similar over the same potential range. This potential-dependent decrease in the 740 cm^−1^ peak area on Au@PtRuNi, while remaining constant on Au@PtRu, indicates that the addition of nickel fundamentally modifies the OH_ad_ adsorption dynamics.

Here, we propose that Ni(OH)_2_ acts as a dual-function modifier. First, it facilitates water activation and supplies additional OH_ad_ species through its inherent oxophilic character; second, it creates favorable interfacial sites at the Ni(OH)_2_–RuO_*x*_ perimeter for efficient OH_ad_ consumption. At lower potentials (−0.015 to 0.235 V *vs.* RHE), the enhanced OH_ad_ generation dominates, resulting in a larger 740 cm^−1^ peak on Au@PtRuNi compared to Au@PtRu. However, at higher potentials (above 0.235 V *vs.* RHE), OH_ad_ is consumed more rapidly through the interfacial Volmer step at Pt–RuO_*x*_ sites promoted by Ni(OH)_2_, causing the 740 cm^−1^ peak to decrease. The potential role of Ni(OH)_2_ will be further discussed in the Discussions section.

## 4. Discussions

The HOR *in situ* SERS results on the Au@PtRuNi catalyst provide clear evidence that Ru/RuO_*x*_ is still the dominant OH_ad_ adsorption site, whereas Ni in Au@PtRuNi is oxidized into hydroxide or oxyhydroxide phases, most likely Ni(OH)_2_, as evidenced by the 566 cm^−1^ peak. The presence of the potential-dependent 566 cm^−1^ peak, as well as the potential-dependent decrease in the 740 cm^−1^ OH_ad_ peak area on Au@PtRuNi, suggests a bifunctional mechanism in which Ru/RuO_*x*_ and Ni(OH)_2_ play distinct yet complementary roles. Lin *et al.* showed that OH_ad_ adsorbed on the surface of RuO_*x*_ has favorable free energy to react with H_ad_ to form H_2_O (Δ*G* ≈ −0.7 eV).^33^ This places RuO_*x*_ close to the optimal OH_ad_ binding region for alkaline HOR.^45,46^ With the bifunctional mechanism, Pt provides sites for the adsorption of hydrogen atoms and forms H_ad_, while RuO_*x*_ supplies optimally bound OH_ad_ that reacts with H_ad_ to form H_2_O. Our SERS observation shows that the position of the 740 cm^−1^ peak remains unchanged upon Ni addition, indicating that the Pt–RuO_*x*_ bifunctional mechanisms remain intact on Au@PtRuNi. In contrast, the emergence and growth of the ∼566 cm^−1^ band on Au@PtRuNi, but not on Au@PtRu, points to an additional, possibly more “upstream” role of Ni (oxy)hydroxides. The potential ranges for Ni(OH)_2_ and NiOOH formation in the literature strongly suggest that Ni in Au@PtRuNi is transformed *in situ* into Ni(OH)_2_.^38,41,44^

Under this hypothesis, the active surface of Au@PtRuNi can be viewed as a Pt–RuO_*x*_–Ni(OH)_2_ ensemble. In our electrochemical experiments, the HOR has higher reaction activity on Au@PtRuNi than on Au@PtRu; our DFT results (Note #2 and Fig. S2 in SI) also show that the Pt–RuO_*x*_–Ni(OH)_2_ ensemble has modestly more favorable Gibbs free energies for OH adsorption and for H_ad_ + OH_ad_ → H_2_O formation at the Ni(OH)_2_/RuO_*x*_ interface, compared to the RuO_*x*_ surface alone. In addition, we performed RT-TDDFTB simulations of four core–shell nanoparticle models: Au_561_, Au_309_@Pt_252_, Au_309_@Pt_216_Ru_36_, and Au_309_@Pt_116_Ni_116_Ru_20_ (Note #3 and Fig. S3 in SI). The results show that alloying the Au surface with Pt, Ru, and Ni does not produce a distinct new visible resonance, but instead broadens and damps the optical response of the Au core. This suggests that the enhanced HOR activity and the appearance of the 566 cm^−1^ Raman band are unlikely to originate from a stronger plasmonic or optical antenna effect. The optimized multi-metal clusters also exhibit significant structural relaxation upon incorporation of Ru and Ni, which may support the formation and stabilization of catalytically relevant species.

The role of Ni(OH)_2_ can be rationalized by the Pt/Ni(OH)_2_ system studied by Markovic's group in both HOR and hydrogen evolution reaction (HER) electrocatalysis.^27,47^ Tian *et al.*'s research also reported a synergistic interaction between RuO_*x*_ and Ni(OH)_2_ in their system.^48^ In both Markovic and Tian's systems, Ni(OH)_2_ acts as a promoter that accelerates water activation and the production of OH_ad_ while Ru/RuO_*x*_ act as active sites for further reaction steps involving OH_ad_. The spectroscopic evidence from the potential-dependent decrease in the 740 cm^−1^ OH_ad_ peak on Au@PtRuNi also validates our proposed multi-site bifunctional mechanism. Rather than simply increasing the total OH_ad_ availability, Ni(OH)_2_ actively regulates the supply-consumption network at the Pt–RuO_*x*_ sites. These results are consistent with DFT predictions of more favorable OH_ad_ reduction energetics at the Ni(OH)_2_/RuO_*x*_ interface. To summarize, we successfully found spectroscopic evidence of Ni(ii) forming *in situ* that correlates with increased HOR activity. Our *in situ* electrochemical SERS results, together with prior work on Pt/Ni(OH)_2_ and Ru–NiO_*x*_ systems, support a multi-site bifunctional mechanism for the HOR on Au@PtRuNi in which Ni(OH)_2_, RuO_*x*_, and Pt play distinct yet complementary roles. In our Pt–RuO_*x*_–Ni(OH)_2_ framework formed *in situ* from the Au@PtRuNi catalyst, Pt provides sites for H adsorption, Ni(OH)_2_ contributes to water activation and the supply of OH_ad_, and RuO_*x*_ provides thermodynamically optimal adsorption sites for OH_ad_ to react with H_ad_ in the Volmer step. The Ni(OH)_2_–Pt and Ni(OH)_2_–RuO_*x*_ perimeters increase the number of interfacial H_ad_/OH_ad_ pairs and establish a coupled OH_ad_ supply and consumption network. Taken together, our results support a multi-site, bifunctional mechanism and highlight a general strategy for future alkaline HOR catalyst design that not only promotes OH adsorption, but also focuses on the synergistic effect of different oxophilic components for coupled supply and consumption of OH_ad_.

## Conflicts of interest

There are no conflicts to declare.

## Supplementary Material

NA-OLF-D6NA00164E-s001

## Data Availability

Data for this article are available on request. Supplementary information (SI) is available. See DOI: https://doi.org/10.1039/d6na00164e.

## References

[cit1] Zhang J., Shen L., Jiang Y., Sun S.-G. (2020). Nanoscale.

[cit2] Jacobson M. Z., Colella W., Golden D. M. (2005). Science.

[cit3] Soleimani A., Dolatabadi S. H. H., Heidari M., Pinnarelli A., Khorrami B. M., Luo Y., Vizza P., Brusco G. (2024). Multiscale Multidiscip. Model. Exp. Des..

[cit4] İncı M. (2022). Sustain. Energy Technol. Assess..

[cit5] Liu Q., Ranocchiari M., van Bokhoven J. A. (2021). Chem. Soc. Rev..

[cit6] Prats H., Chan K. (2021). Phys. Chem. Chem. Phys..

[cit7] Hasannaeimi V., Mukherjee S. (2019). Sci. Rep..

[cit8] Mu X., Liu S., Chen L., Mu S. (2023). Small Struct..

[cit9] Campos-Roldàn C. A., Alonso-Vante N. (2019). Electrochem. Energy Rev..

[cit10] Weber D., Dosche C., Oezaslan M. (2021). J. Mater. Chem. A.

[cit11] Bouho F. K., Rafaïdeen T., Napporn T. W., Coutanceau C. (2024). Electrochim. Acta.

[cit12] Truong V. M., Tolchard J. R., Svendby J., Manikandan M., Miller H. A., Sunde S., Yang H., Dekel D. R., Barnett A. O. (2020). Energies.

[cit13] Lian Y. (2025). MATEC Web Conf..

[cit14] Osmieri L., Pezzolato L., Specchia S. (2018). Curr. Opin. Electrochem..

[cit15] Hyun J., Kim H.-T. (2023). Energy Environ. Sci..

[cit16] Su L., Wu H., Zhang S., Cui C., Zhou S., Pang H. (2025). Adv. Mater..

[cit17] Zhang X., Xiao X., Chen J., Liu Y., Pan H., Sun W., Gao M. (2022). Energy Environ. Sci..

[cit18] An L., Zhao T., Lei W., Yang C., Yang J., Wang D. (2025). eScience.

[cit19] Fang Y., Wei C., Bian Z., Yin X., Liu B., Liu Z., Chi P., Xiao J., Song W., Niu S., Tang C., Liu J., Ge X., Xu T., Wang G. (2024). Nat. Commun..

[cit20] Huang Z., Hu S., Sun M., Xu Y., Liu S., Ren R., Zhuang L., Chan T.-S., Hu Z., Ding T., Zhou J., Liu L., Wang M., Huang Y., Tian N., Bu L., Huang B., Huang X. (2024). Nat. Commun..

[cit21] Liao Y., Wang S., Zhang Y., Zhang Y., Gao Y., Mu X., Liu S., Wang D., Dai Z. (2023). Adv. Sens. Energy Mater..

[cit22] Gannon W. J. F., Dunnill C. W. (2019). Electrochim. Acta.

[cit23] Shah A. H., Zhang Z., Huang Z., Wang S., Zhong G., Wan C., Alexandrova A. N., Huang Y., Duan X. (2022). Nat. Catal..

[cit24] Li J., Ghoshal S., Bates M. K., Miller T., Davies V., Stavitski E., Attenkofer K., Mukerjee S., Ma Z.-F., Jia Q. (2017). Angew. Chem., Int. Ed..

[cit25] Qiu Y., Xie X., Li W., Shao Y. (2021). Chin. J. Catal..

[cit26] Li W., Feaster J. T., Akhade S. A., Davis J. T., Wong A. A., Beck V. A., Varley J. B., Hawks S. A., Stadermann M., Hahn C., Aines R. D., Duoss E. B., Baker S. E. (2021). ACS Sustain. Chem. Eng..

[cit27] Strmčnik D., Uchimura M., Wang C., Subbaraman R., Danilovic N., Vliet D. V., Paulikas A. P., Stamenković V. R., Marković N. M. (2013). Nat. Chem..

[cit28] Shen L., Lu B.-A., Qu X., Ye J., Zhang J., Yin S., Wu Q.-H., Wang R., Shen S.-Y., Sheng T., Jiang Y., Sun S.-G. (2019). Nano Energy.

[cit29] Feng Y., Lu S., Fu L., Yang F., Feng L. (2023). Chem. Sci..

[cit30] Chen X., Wang X., Le J.-B., Li S., Wang X., Zhang Y., Radjenovic P. M., Zhao Y., Wang Y., Lin X.-M., Dong J.-C., Li J. (2023). Nat. Commun..

[cit31] Negahdar L., Parlett C. M. A., Isaacs M. A., Beale A. M., Wilson K., Lee A. F. (2020). Catal. Sci. Technol..

[cit32] Wang R., Li D., Maurya S., Kim Y. S., Wu Y. A., Liu Y., Strmčnik D., Marković N. M., Stamenković V. R. (2019). Nanoscale Horiz..

[cit33] Lin X.-M., Wang X., Deng Y.-L., Chen X., Chen H.-N., Radjenovic P. M., Zhang X.-G., Wang Y., Dong J.-C., Tian Z.-Q., Li J. (2022). Nano Lett..

[cit34] Wang Y., Wang X.-T., Ze H., Zhang X.-G., Radjenovic P. M., Zhang Y.-J., Dong J.-C., Tian Z.-Q., Li J. (2020). Angew. Chem., Int. Ed..

[cit35] Zhang H., Duan S., Radjenovic P. M., Tian Z.-Q., Li J.-F. (2020). Acc. Chem. Res..

[cit36] Brust M., Fink J., Bethell D., Schiffrin D. J., Kiely C. J. (1995). J. Chem. Soc. Chem. Commun..

[cit37] Frens G. (1973). Nat. Phys. Sci..

[cit38] Oshchepkov A. G., Braesch G., Bonnefont A., Savinova E. R., Chatenet M. (2020). ACS Catal..

[cit39] Hall D. S., Lockwood D. J., Bock C., MacDougall B. R. (2015). Proc. R. Soc. A.

[cit40] Hall D. S., Bock C., MacDougall B. R. (2013). J. Electrochem. Soc..

[cit41] Alsabet M., Grdeń M., Jerkiewicz G. (2015). Electrocatalysis.

[cit42] Yeo B. S., Bell A. T. (2012). J. Phys. Chem. C.

[cit43] Hall D. S., Lockwood D. J., Poirier S., Bock C., MacDougall B. R. (2012). J. Phys. Chem. A.

[cit44] Xue Y., Shi L., Liu X., Fang J., Wang X., Setzler B. P., Zhu W., Yan Y., Zhuang Z. (2020). Nat. Commun..

[cit45] Men Y., Tan Y., Li P., Jiang Y., Li L., Su X., Men X., Sun X., Chen S., Luo W. (2024). Angew. Chem., Int. Ed..

[cit46] Yang C., Li Y., Ge C., Jiang W., Cheng G., Zhuang L., Luo W. (2022). Chin. J. Chem..

[cit47] Danilovic N., Subbaraman R., Strmcnik D., Chang K., Paulikas A. P., Stamenkovic V. R., Markovic N. M. (2012). Angew. Chem., Int. Ed..

[cit48] Tian J., Wang M., Xie J., Hu J., Lu Z., Cao Y. (2025). Fuel.

