# Supplementary material for: In situ SERS reveals nickel hydroxide formation in PtRuNi catalysts enhances hydrogen oxidation
Source: Nanoscale Adv. 2026 May 15;8(13):3761–8. doi: 10.1039/d6na00164e (PMC13234769; doi:10.1039/d6na00164e)
Supplement: NA-008-D6NA00164E-s001 [file NA-008-D6NA00164E-s001.pdf]

Supplementary Information (SI) for Nanoscale Advances.
This journal is © The Royal Society of Chemistry 2026

Supplementary Information

In-situ SERS Reveals Nickel Hydroxide Formation in PtRuNi Catalysts Enhancing Hydrogen Oxidation

Kai Tao[a], Mingxiao Han[b], Li Jiang[b] ,Shangzhong Jin[b], Tommaso Giovannini[c], Denis Garoli[b,d*], Zhefei Zhao[e], Qiang Lin[f], Huaizhou Jin[a*]

[a] Key Laboratory of Quantum Precision Measurement, College of Physics, Zhejiang University of Technology, Hangzhou 310014, China.

[b] College of Optical and Electronic Technology, China Jiliang University, Hangzhou 310018, China.

[c] Department of Physics, University of Rome Tor Vergata, and INFN, Via della Ricerca Scientifica 1, I-00133, Rome, Italy

[d] Dipartimento di Scienze e Metodi dell'Ingegneria, Università degli Studi di Modena e Reggio Emilia, Viale Amendola 2, Reggio Emilia (Italy)

[e] Department of Applied Chemistry, Petroleum and Chemical Industry Key Laboratory of Organic Electrochemical Synthesis, State Key Laboratory of Green Chemical Synthesis and Conversion, Zhejiang University of Technology, Hangzhou 310014, China.

[f] State Key Laboratory of Ocean Sensing & Institute of Quantum Sensing & School of Physics, Zhejiang University, Hangzhou, 310058, China

Corresponding authors: Huaizhou Jin - jinhuaizhou@zjut.edu.cn; Prof. Denis Garoli – denis.garoli@unimore.it

Note #1. Additional SERS analysis – peak fitting.

Supplementary Figure S1. Peak fitting results for 740cm^{-1}; (a) area under 740cm^{-1} peak for HOR at Au@PtRuNi nanoparticles under potentials from -15 to 535mV; (b) area under 740cm^{-1} peak for HOR at Au@PtRu nanoparticles under potentials from -15 to 535mV

Note #2. DFT simulation computation details

DFT calculations were performed to investigate HOR on both Pt-RuO_x and Pt-RuO_x-$Ni(OH)_2$ structures using the Vienna Ab Initio Simulation Package (VASP) [1,2]. The generalized gradient approximation (GGA) with the Perdew–Burke–Ernzerhof (PBE) exchange–correlation functional was employed. The projector augmented wave (PAW) method was used to describe the interaction between valence electrons and ionic cores, with a plane-wave kinetic energy cutoff of 450 eV. To model the catalyst surface, a Pt(111) slab was constructed and expanded into a 6 × 6 supercell, with a vacuum layer of 15 Å along the z-direction to avoid spurious interactions between periodic images. RuO_x and RuO_x–$Ni(OH)_2$ nanoclusters were then placed on the Pt(111) surface to represent the RuO_x and RuO_x–$Ni(OH)_2$ structures formed during catalysis on Au@PtRu and Au@PtRuNi catalysts.

Geometric optimizations were carried out using the conjugate-gradient algorithm until the total energy and atomic forces converged to 1.0×10^{-5} eV and 4.0×10^{-2} eV Å^{-1}, respectively. During structure relaxation, a Γ-centered 2 × 2 × 1 k-point mesh was used to sample the Brillouin zone. To account for dispersion interactions between the surface models and adsorbates, Grimme's DFT-D3 correction was applied [3]. The final, optimized structures Pt-RuO_x and Pt-RuO_x-$Ni(OH)_2$ are shown in Supplementary Figure S2 (a) and (b).

For the adsorption of OH and the subsequent $1/2H_2 + OH_{ad} \rightarrow H_2O$ step, Gibbs free energy changes (ΔG) were calculated by:

$$\Delta G = \Delta E + \Delta E_{ZPE} - T\Delta S$$

where ΔE is the electronic energy difference between the free-standing and adsorption states of reaction intermediates; ΔE_{ZPE} and ΔS are the changes in zero-point energies and entropy, respectively, which were obtained from the vibrational frequency calculations; T is the temperature, and was set to be 298.15 K in this work.

The resulting ΔG for the two relevant HOR steps on Pt-RuO_x-$Ni(OH)_2$ and Pt-RuO_x are shown in Supplementary Figure S2 (c). The results show that in the presence of $Ni(OH)_2$, OH adsorption at the heterojunction becomes more favorable (-2.71eV vs -2.58eV), and the reaction free energy for H_2O formation step is also improved (-0.838eV vs -0.732eV). Both steps in HOR are improved, and these results are consistent with the hypothesis that interfacial $Ni(OH)_2$ promotes OH_{ad} formation and consumption at the Pt–RuO_x ensemble, in line with the bifunctional mechanism inferred from our SERS experiments.

Supplementary Figure S2. (a) and (b) structure of RuO_x nanocluster and $Ni(OH)_2$-RuO_x nanoclusters on Pt (111), respectively; elements in the structure are blue-Pt; cyan-Ru; purple-Ni; red-O; gray-H; (c) Gibbs free energy results of HOR's OH adsorption and Volmer step on both structures.

Note #3. Real-Time Time Dependent Density Functional Tight Binding simulations

The atomistic models of the investigated nanoparticles for Density Functional Tight Binding (DFTB) calculations were generated starting from Mackay six-shell icosahedral clusters (561 atoms). The initial Au geometry was built using the experimental Au lattice constant of 4.0782 Å. Four core-shell nanoparticle models were considered: Au_{561}, $Au_{309}@Pt_{252}$, $Au_{309}@Pt_{216}Ru_{36}$, and $Au_{309}@Pt_{116}Ni_{116}Ru_{20}$. For the alloyed systems, the 309 Au inner atoms were kept as Au, whereas the atoms belonging to the outer shell were replaced by Pt, Ru, and Ni according to the target compositions, with specific ratio (Pt:Ru – 6:1; Pt:Ni:Ru – 6:6:1). The distribution of Pt, Ru, and Ni atoms on the external shell was generated randomly.

Geometry optimizations were performed with DFTB+ software [4] using the SCC-DFTB Hamiltonian [5] and the PTBP Slater–Koster parameter set [6]. The maximum angular momentum was set to d for Au, Pt, Ru, and Ni. Electronic occupations were described using Fermi smearing at 300 K. SCC convergence was controlled using a Broyden mixer with a mixing parameter of 0.05, an SCC tolerance of 10^{-5}. For the alloyed core–shell nanoparticles, geometry optimization was carried out using the conjugate-gradient driver. The Au309 core was kept fixed. The optimization was stopped the maximum force component was below 0.05 a.u. After optimization, Au_{561} and $Au_{309}@Pt_{252}$ retained an icosahedral shape, whereas the inclusion of the third and fourth metals in $Au_{309}@Pt_{216}Ru_{36}$ and $Au_{309}@Pt_{116}Ni_{116}Ru_{20}$ induced a more substantial structural rearrangement of the external shell (see Supplementary Fig. S3a).

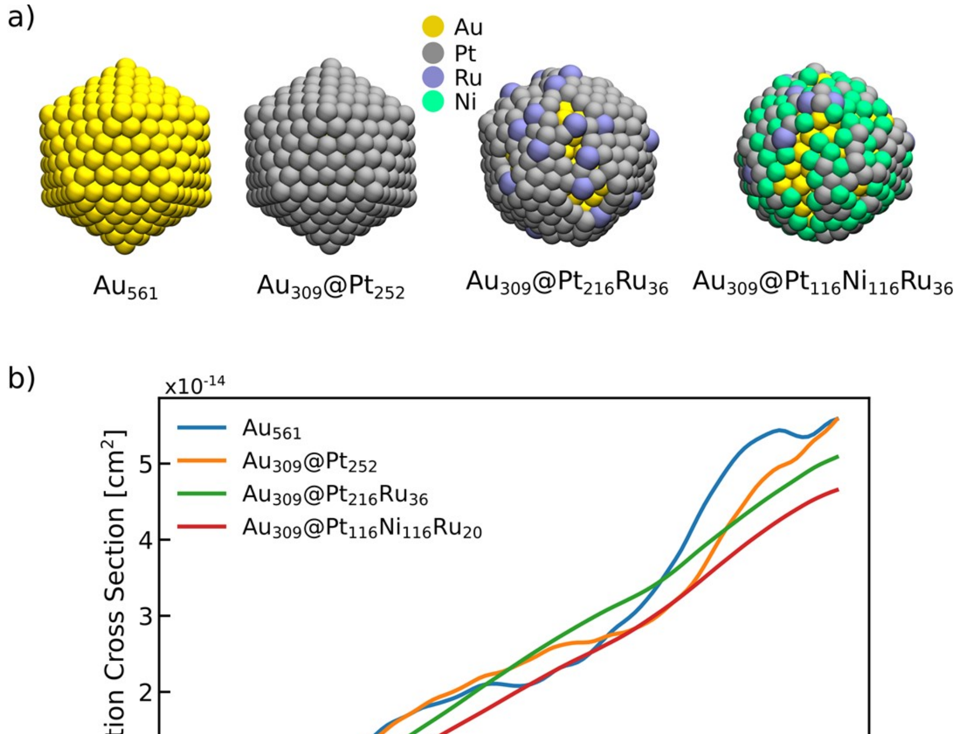

Supplementary Figure S3. (a) Optimized structures of Au_{561}, $Au_{309}@Pt_{252}$, $Au_{309}@Pt_{216}Ru_{36}$ and $Au_{309}@Pt_{116}Ni_{116}Ru_{36}$ nanoparticles. (b) Isotropic absorption cross section as a function of the chemical composition of the nanoclusters computed at the RT-TDDFTB level.

Optical absorption spectra were computed at the real-time time-dependent DFTB (RT-TDDFTB) level [7,8]. Starting from the optimized geometries, the systems were subjected to a delta-kick electric-field perturbation along the three directions. Each real-time propagation was performed for 8000 steps with a time step of 0.4 a.u. (~77 fs). The field strength used for the kick perturbation was 0.001 V $Å^{-1}$. The absorption spectrum was obtained from the Fourier transform of the induced dipole moment and then isotropically averaged. An exponential damping corresponding to a Lorentzian broadening with a full width at half maximum of 0.30 eV was applied.

The calculated absorption spectra are given in Supplementary Fig. S3b. Although the considered systems are small, the comparison between the four chemical compositions reveals some clear trends. First, the inclusion of Pt, Ru, and Ni in the external shell does not generate a distinct new optical resonance in the visible region. Instead, the optical response predicted for Au_{561} is progressively broadened and, in some cases, damped upon alloying the surface with the catalytically active metals. The results therefore support the interpretation that the Au core mainly acts as the plasmonic antenna required for optical and SERS activity, whereas the Pt, Ru, and Ni atoms in the shell primarily control the surface chemistry relevant to the catalytic process.

The $Au_{309}@Pt_{116}Ni_{116}Ru_{20}$ NP, which corresponds to the experimentally most active catalyst, is not associated with the strongest optical response in the spectral region relevant to the SERS experiment. This suggests that the enhanced catalytic activity of Au@PtNiRu and the

emergence of the 566 cm^{-1} Raman band cannot rationalized in terms of a stronger optical antenna effect. Indeed, our results support the interpretation that the experimentally observed changes originate mainly from modifications of the surface chemistry. The structural behavior of the optimized models also supports this picture. While Au_{561} and $Au_{309}@Pt_{252}$ retain a largely icosahedral morphology, the introduction of Ru and especially the simultaneous presence of Pt, Ru, and Ni lead to a more pronounced rearrangement of the external shell. This suggests that the multimetallic shell is not merely a passive optical coating on the Au core, but can undergo significant structural relaxation, which can affect the formation and stabilization of catalytically relevant species.

References

1 G. Kresse and J. Furthmüller, *Comput. Mater. Sci.*, 1996, **6**, 15–50.

2 G. Kresse and J. Furthmüller, *Phys. Rev. B*, 1996, **54**, 11169.

3 S. Grimme, J. Antony, S. Ehrlich and H. Krieg, *J. Chem. Phys.*, 2010, **132**, 154104.

4 B. Hourahine, B. Aradi, V. Blum, F. Bonafé, A. Buccheri, C. Camacho, C. Cevallos, M. Y. Deshaye, T. Dumitrică, A. Dominguez, S. Ehlert, M. Elstner, T. van der Heide, J. Hermann, S. Irle, J. J. Kranz, C. Köhler, T. Kowalczyk, T. Kubař, I. S. Lee, V. Lutsker, R. J. Maurer, S. K. Min, I. Mitchell, C. Negre, T. A. Niehaus, A. M. N. Niklasson, A. J. Page, A. Pecchia, G. Penazzi, M. P. Persson, J. Řezáč, C. G. Sánchez, M. Sternberg, M. Stöhr, F. Stuckenberg, A. Tkatchenko, V. W. -z. Yu and T. Frauenheim, *J. Chem. Phys.*, 2020, **152**, 124101.

5 M. Elstner, D. Porezag, G. Jungnickel, J. Elsner, M. Haugk, Th. Frauenheim, S. Suhai and G. Seifert, *Phys. Rev. B*, 1998, **58**, 7260–7268.

6 M. Cui, K. Reuter and J. T. Margraf, *J. Chem. Theory Comput.*, 2024, **20**, 5276–5290.
7 F. P. Bonafé, B. Aradi, B. Hourahine, C. R. Medrano, F. J. Hernández, T. Frauenheim and C. G. Sánchez, *J. Chem. Theory Comput.*, 2020, **16**, 4454–4469.
8 O. A. Douglas-Gallardo, M. Berdakin, T. Frauenheim and C. G. Sánchez, *Nanoscale*, 2019, **11**, 8604–8615.
